# Drug Screening of Flavonoids as Potential VEGF Inhibitors Through Computational Docking and Cell Models

**DOI:** 10.3390/molecules30020257

**Published:** 2025-01-10

**Authors:** Shengying Lin, Roy Wai-Lun Tang, Yutong Ye, Chenxi Xia, Jiahui Wu, Ran Duan, Ka-Wing Leung, Tina Ting-Xia Dong, Karl Wah-Keung Tsim

**Affiliations:** 1 Center for Chinese Medicine, Division of Life Science, The Hong Kong University of Science and Technology, Clear Water Bay, Kowloon, Hong Kong, China; lishlin@ust.hk (S.L.); roytwl@ust.hk (R.W.-L.T.); yyebl@connect.ust.hk (Y.Y.); chenxi.xia@connect.ust.hk (C.X.); jwuct@connect.ust.hk (J.W.); duanran@ust.hk (R.D.); lkwing@ust.hk (K.-W.L.); botina@ust.hk (T.T.-X.D.); 2 State Key Laboratory of Molecular Neuroscience, Division of Life Science, The Hong Kong University of Science and Technology, Clear Water Bay, Kowloon, Hong Kong, China

**Keywords:** computational docking, drug screening, flavonoids, VEGF inhibitor, angiogenesis

## Abstract

Vascular endothelial growth factor (VEGF), also known as VEGF-A, has been linked to various diseases, such as wet age-related macular degeneration (wAMD) and cancer. Even though there are VEGF inhibitors that are currently commercially available in clinical applications, severe adverse effects have been associated with these treatments. There is still a need to develop novel VEGF-based therapeutics against these VEGF-related diseases. Here, we established a series of VEGF-based computational docking analyses and cell models, such as a wound healing assay in HaCaT cells and an evaluation of NF-κB performance in macrophages, to screen a large library of flavonoid-type phytochemicals. Three flavonoids, namely, farrerol, ononin and (−)-epicatechin, were shown to express binding affinities to VEGF protein and inhibit VEGF-mediated biological activities. The investigation evidently suggested that the three flavonoids above could be considered potential anti-VEGF agents for the following drug development against VEGF-mediated diseases.

## 1. Introduction

Vascular endothelial growth factor (VEGF), also known as VEGF-A, is a member of the VEGF family, which has been known to play a central role in angiogenesis. Generally, the VEGF family is a group of proteins consisting of 8 cysteine species connected via a cysteine knot motif, and each member expresses their biological activities by binding to their corresponding receptors [1,2]. Structurally, VEGF contains three intra-molecular disulfides within each of the polypeptide chains and exists as a covalent homodimeric protein. Functionally, VEGF is recognized as an inducer in driving embryonic and somatic angiogenesis [2,3].

For decades, VEGF was thought to be specifically related to endothelial cells due to its crucial roles in mitogenesis and chemotaxis. Nevertheless, several lines of evidence have revealed that VEGF exerts fundamental biological functions among non-endothelial cells [4,5,6]. For example, VEGF regulates monocyte chemotaxis within monocytes by binding to its corresponding receptor-VEGFR-1, and this VEGF/VEGFR-1 complex is able to mediate cell proliferation, cell migration, cell adhesion, etc., in HaCaT cells (human keratinocytes). It is also known that the overexpression of VEGF has been linked to several diseases, such as wet age-related macular degeneration (wAMD) and cancer [4,5,6].

Flavonoids are a type of phytochemicals consisting of polyphenolic structures and are commonly found in natural products, including vegetables and fruits. Generally, flavonoids contain a variety of medicinal features, such as anti-inflammatory, antioxidant, neuroprotective and cardioprotective effectiveness [7]. Intriguingly, flavonoids are associated with angiogenesis and VEGF-related disease [8,9]. These inspired us to conduct an intensive drug screening of flavonoid-type natural compounds that could be utilized as potent anti-VEGF and anti-angiogenesis inhibitors for subsequent drug development against VEGF-mediated disease.

Computational docking has been an efficient in silico tool utilized in the initial phase of drug discovery [10]. The virtual screening often commences with determining the protein binding domain that is responsible for small ligand binding and allows the detection of molecules with high affinity at the targeted protein. The goal of such an analysis is to locate a subset of compounds out of a large library for the following biological evaluations. Here, we employed SEESAR software v. 13.0 as a computational tool with an aim to screen thousands of flavonoid-type phytochemicals, of which farrerol, ononin and (−)-epicatechin showed decent binding affinities to VEGF protein and promising anti-VEGF potency through multiple rounds of biological evaluations. The results served as evidence that the three phytochemicals above could be considered potent anti-VEGF inhibitors for the following drug development.

## 2. Results

### 2.1. Computational Analysis-Aided Drug Screening of Flavonoids Against VEGF Protein

Initially, we employed SEESAR as computational software for the molecular docking analysis of flavonoid-type phytochemicals. Axitinib was identified as a selective tyrosine kinase inhibitor targeting VEGF/VEGFR-1 [11] and was used as a positive control that displayed decent affinity to the protein with a binding energy of −17.4 kJ/mol. Out of more than 1000 compounds being screened, the top 20 ligands with low binding energies are summarized in Table S1 (Supplementary Document S1).

Subsequently, several parameters were utilized to outline the ADMET profile of each phytochemical. According to Lipinski’s “Role of Five”, a compound with a molecular weight lower than 500 Da and a Log *p* value lower than 5 shows relatively high drug-likeness [12]. In addition, TPSA is linked to molecular lipophilicity, aqueous solubility and intestinal permeability. Interestingly, the drug-like medicinal ligands generally have TPSA values lower than 140 Å^2^ [13]. The Human Intestinal Absorption (HIA) descriptor represents the classification of absorption, and a positive HIA descriptor indicates over 30% intestinal absorption after drug administration [14]. The blood–brain barrier (BBB) and *hERG* are associated with neurotoxicity and cardiotoxicity, respectively, and compounds being screened at this stage are expected to be absorbed without penetrating the BBB or interacting with *hERG* [15,16]. P-gp is identified as an ATP-driven efflux pump and is responsible for active transportation, which could lead to a reduction in bioavailability [17]. Taking these factors into account, three flavonoid-type molecules, i.e., farrerol, ononin and (−)-epicatechin, were selected for the subsequent evaluations (Table S1, Supplementary Document S1).

**Figure 1:**
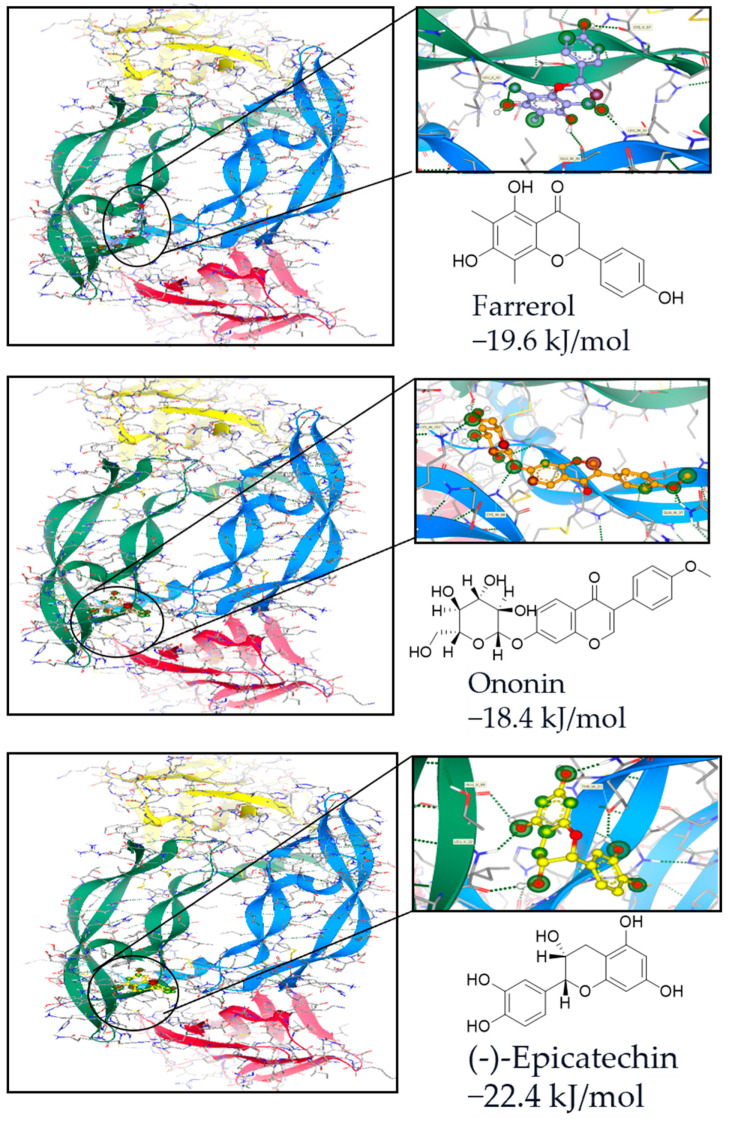
Computational docking analysis of farrerol, ononin and (−)-epicatechin against VEGF protein. Protein structure was downloaded from the Protein Data Bank (PDB code: 1FLT), and chemical structures were obtained from Chemdraw software v. 20.0. Axitinib was used as a positive control, and the binding energy was −17.4 kJ/mol.

Intriguingly, the three phytochemicals were predicted to have lower binding energies than the positive control—Axitinib. This suggested that the three flavonoids are likely to act as VEGF inhibitors by disrupting the complex between VEGF and its receptor (Figure 1). As shown in Figure S1 (Supplementary Document S2), farrerol, ononin and (−)-epicatechin were found to establish fundamental protein–ligand interactions with the VEGF protein. Compared with axitinib, which interacted with Val14, Asp19, Asn75 and Thr79, farrerol formed hydrogen bonds with Ile 29, Glu30, Leu32 and Cys57, while ononin interacted with Gln37, Gly59, Cys61 and Lys107. Moreover, (−)-epicatechin bound to the same binding pocket and established hydrogen bonds with Glu 30, Thr 31, Leu 32 and Cys57.

Chemical degradation has been a major factor resulting in molecular hydrolysis, oxidation, light-catalyzed degradation and so on [18]. Therefore, stability in aqueous solution, including acidic, neutral and basic conditions, has become a crucial requirement for a promising drug candidate. Prior to the biochemical assay, three phytochemicals were subjected to HPLC analysis to determine their stabilities in various solutions. Glycine buffer (pH 8–11), PBS (pH 7–8) and acetate buffer (pH 4–6) were employed to cover the main pH ranges. No major degradation was observed from all three chemical samples in acidic, neutral and basic conditions (RSD < 10%), although some mild degradation of (−)-epicatechin was induced in acidic and basic environments (Table 1). This indicated that farrerol and ononin were relatively stable in diverse aqueous conditions, and a neutral condition might be required when (−)-epicatechin is stored and transported.

**Figure 2:**
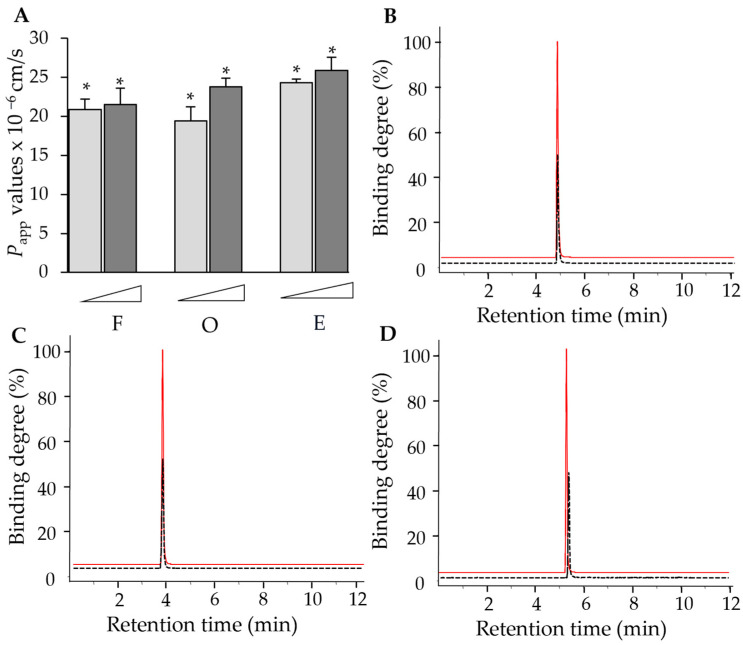
(**A**) Farrerol, ononin and (−)-epicatechin showed cell absorption in Caco-2 cells at concentrations of 1 µM and 10 µM. Caco-2 cells were cultured until fully differentiated after being cultured for 21 days, and the integrity of cell monolayer was determined by the transepithelial electrical resistance (TEER). * *p* < 0.05. Binding degrees of farrerol (**B**), ononin (**C**) and (−)-epicatechin (**D**) were calculated through the following equation: binding degree = (C_pre_ − C_post_)/C_pre_, where C_pre_ was the initial concentration of the tested ligand and C_post_ was the unbound chemical in the filtrate. Red curve: initial concentration of chemicals; black curve: concentration of chemicals binding to the protein (C_pre_ − C_post_). *n* = 4.

The Caco-2 cell assay was used to evaluate the absorption of phytochemicals after administration. The positive control, propranolol, was identified as a well-absorbed agent in clinical application and displayed a permeability coefficient (*P_app_*) value of 46.8 × 10^−6^ cm/s in our initial attempt [19]. In the following trials, we revealed that the three flavonoid-type compounds generally showed *P_app_* values between 19 × 10^−6^ and 26 × 10^−6^ cm/s (Figure 2A). According to Ahmed et al., these flavonoids were likely to exhibit good absorptions and oral bioavailability [19], which was in agreement with our in silico prediction above.

An ultrafiltration assay was utilized to determine the protein binding affinity between the tested compounds and the VEGF protein. Compared with initial samples without the presence of the protein, the protein binding degrees for farrerol, ononin and (−)-epicatechin at a concentration of 0.02 µM were determined as 58.3%, 53.4% and 50.3%, respectively (Figure 2B–D). This implied that the three flavonoids were able to display robust binding performances to the VEGF protein and, therefore, regulate the downstream biological activities.

### 2.2. Phytochemicals Suppressed VEGF-Regulated Biological Activities

**Figure 3:**
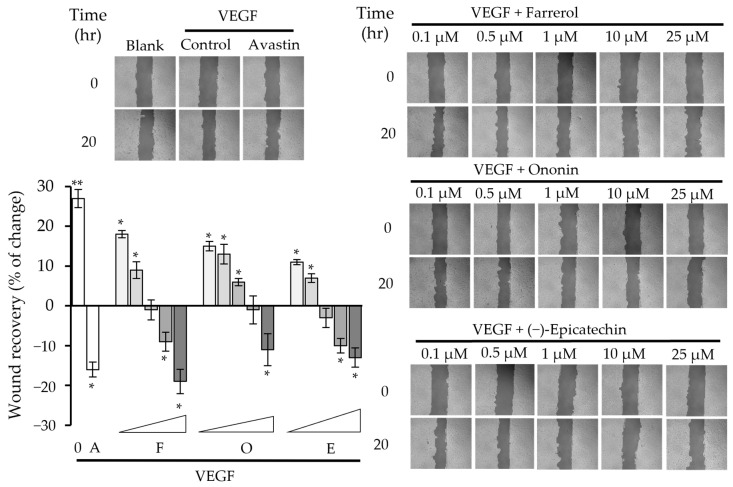
Wound healing evaluation of farrerol, ononin and (−)-epicatechin. Photos of each cell scrape at 0 (At_0_) and 20 h (At_20_) were captured with microscope and imaging software (Zen, https://www.zeiss.com/microscopy/en/home.html, accessed on 1 September 2024) at a 10× magnification. The data indicate the mean ± SD (*n* = 4) fold change compared with the blank group, and the asterisks represent statistically significant differences such that * *p* < 0.05 and ** *p* < 0.01 compared with the blank group. A: positive control, Avastin; F: farrerol; O: ononin; E: (−)-epicatechin.

At the beginning of in vitro biological evaluations, an MTT assay was employed to detect the cytotoxic effects of each flavonoid-type phytochemical. As shown in Figure S2 (Supplementary Document S2), cells remained unaffected when being treated with farrerol, ononin and (−)-epicatechin separately at various concentrations up to 100 µM, suggesting that the three chemicals were unlikely to induce significant apoptosis within the cell line. A wound recovery assay in the HaCaT cell line was employed to evaluate the anti-VEGF efficacy of the selected flavonoids. VEGF was found to promote wound healing rate, and avastin, as a positive control, suppressed the wound recovery that was induced by VEGF protein. As anticipated, farrerol, ononin and (−)-epicatechin were able to express inhibition to the wound recovery rate with IC_50_ values of 4.7 µM, 3.8 µM and 9.2 µM, respectively (Figure 3). This implied that the three flavonoids could suppress the VEGF protein and disrupt VEGF-induced biological activity, which was in line with our observations from in silico and protein assays.

**Figure 4:**
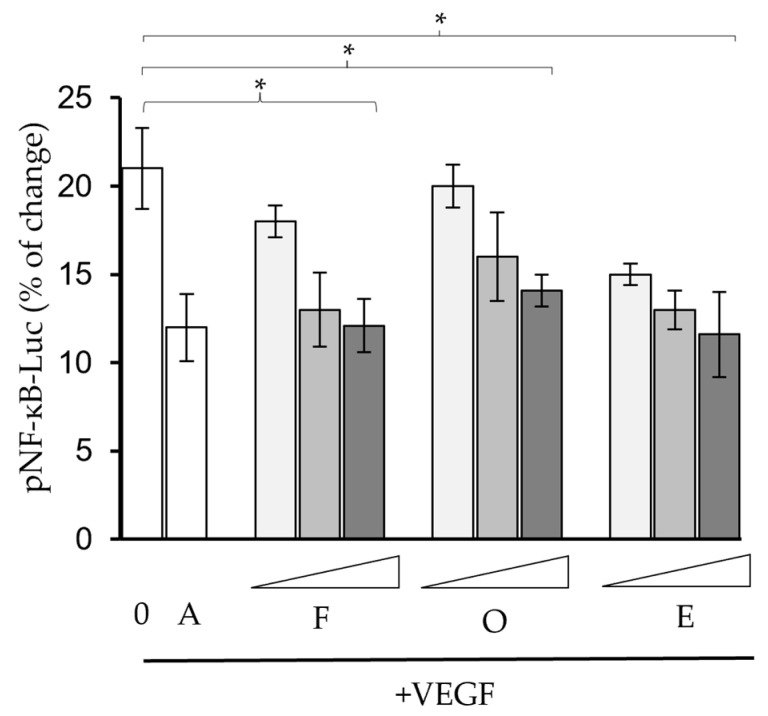
Farrerol, ononin and (−)-epicatechin attenuated VEGF-induced NF-κB activities. The pNF-κB constructs were transported by jetPRIME (Polyplus, Illkirch-Graffenstaden, France) reagent on the fourth day, and the chemicals were tested at concentrations of 1 µM, 10 µM and 25 µM. A: avastin (10 ng/mL). *n* = 4. * *p* < 0.05, compared with the blank group. A: positive control, Avastin; F: Farrerol; O: ononin; E: (−)-epicatechin.

In parallel, we employed a cell assay to detect if VEGF was able to induce a downstream NF-κB signal and whether the phytochemicals could inhibit such a VEGF-regulating signal pathway. It has been previously revealed that VEGF activates the NF-κB signal and inflammation-mediated angiogenesis [20,21]. Indeed, we identified that NF-κB activity was enhanced in the presence of the VEGF protein, and avastin was able to attenuate the NF-κB signal by inhibiting VEGF. In our following attempts, it was observed that when the three flavonoids were introduced to the VEGF-containing mixture, NF-κB activities were suppressed in dose-dependent manners. This indicated that farrerol, ononin and (−)-epicatechin could reduce the activation of NF-κB through the disruption of the VEGF protein (Figure 4).

## 3. Discussion

VEGF/VEGFR-1 signaling plays a crucial role in regulating various biological activities and diseases [22]. Fong et al. conducted intensive in vivo studies in which gene *VEGFR-1* in mice was knocked out, and they observed that the VEGF/VEGFR-1 complex was robustly blocked, leading to dysfunction of blood vessels and the death of mice [23]. This served as strong evidence that VEGF is a fundamental factor in regulating angiogenesis by binding to its receptor VEGFR-1. Furthermore, VEGF is also responsible for diseases other than wAMD and cancer. For instance, pre-eclampsia was triggered in 5–7% of pregnant women, and this usually resulted in hypertension and proteinuria within mothers as well as growth retardation within the fetus. Interestingly, the VEGF/VEGFR-1 complex is significantly overexpressed and present at a high level among pre-eclampsia patients [24,25]. Taken together, VEGF-based therapy was recognized as a considerable strategy in drug development against various diseases.

There have been several VEGF inhibitors approved by the FDA and currently utilized in clinical practices, and the first class of these was monoclonal antibodies [26]. Bevacizumab (avastin) was the first monoclonal antibody targeting the VEGF protein and preventing the kinase from being associated with its receptors. However, Bevacizumab was reported to cause cardiotoxic effects, and patients were recommended to discontinue the treatment when such a side effect occurred [27]. As for small molecule inhibitors, pazopanib and sunitinib are antagonists against multiple VEGF receptors, such as VEGFR-1 and VEGFR-2, while sorafenib was identified as a pan-kinases inhibitor against CRAF, BRAF, VEGF, VEGFR-2 and so on. Nevertheless, the risks of arrhythmias, heart failure and cardiac ischemia were detected in patients after the administration of these three drugs [2,28]. In short, adverse effects were observed from currently marketed treatments in clinical application, and this suggested that there is an urgent need to develop more VEGF-targeted therapeutics with higher efficacy and lower toxicity.

Previously, extensive investigations have been conducted by our team with the aim to discover potent phytochemicals as potential anti-VEGF inhibitors. Specifically, HerboChips technology was utilized to screen a large number of traditional Chinese medicine (TCM) herbal extracts and TCM-derived natural molecules. As a result, out of thousands of samples we screened, resveratrol and polydatin were found to express robust inhibition to VEGF-mediated biological activities, including cell proliferation and migration in endothelial cells and phosphorylation of downstream signaling (PI3-K/Akt, JNK and eNOS) [29]. Furthermore, in another animal study, it was observed that resveratrol could significantly suppress tumor growth in nude mice and trigger synergistic anti-VEGF efficacy when in combination with ginkgetin [30]. In the following docking analysis, resveratrol and polydatin were shown to bind to the VEGF protein with binding energies of −19.1 kJ/mol and −11.4 k/mol, respectively.

Flavonoids have been utilized as the main treatment against various diseases for many years due to their diverse biological functions as well as their safety record in clinical applications. It has been widely reported that flavonoids expressed significant inhibitory activities to angiogenesis and regulated VEGF-related diseases [31,32]. For instance, scutellarin was found to disrupt tumor-associated angiogenesis by reducing transcription factor AP-1, while chrysin contained remarkable anti-angiogenesis properties through inhibiting VEGF/VEGFR2 expression and the phosphorylation of factors, e.g., JAK1 and STAT3 in HUVECs [31,32,33]. In this study, we revealed that three flavonoid-type phytochemicals, farrerol, ononin and (−)-epicatechin, expressed remarkable inhibition to the VEGF protein and VEGF-mediated biological activities. Interestingly, the chemical scaffolds of these three phytochemicals have been previously reported to contain fundamental biological effectiveness. For instance, several isocoumarin derivatives shared a similar chemical fragment with farrerol as well as (−)-epicatechin and showed significant inhibition to inflammation and cancer cells, while a group of ononin-type analogs, 3-glycosylated isocoumarins, were synthesized and expressed a reduction in blood sugar level [34,35]. This evidently indicated that the three flavonoids, in particular, their chemical scaffolds, are likely to display promising biological activities and could be considered for further therapeutic applications.

## 4. Materials and Methods

### 4.1. Materials

Dulbecco’s modified Eagle medium (DMEM), fetal bovine serum (FBS) and other related reagents utilized herein were purchased from Thermo Fisher Scientific (Waltham, MA, USA). Antibodies were purchased from Cell Signaling Technology (CST; Beverly, MA, USA). MTT [3-(4,5 dimethy-2-thiazolyl)-2,5-diphenyl-2*H*-tetrazolium bromide] and the DCFH-DA probe (20,70-dichlorofluorescein diacetate) were purchased from Sigma-Aldrich (St. Louis, MO, USA). All phytochemicals in this report were obtained from Chengdu Herbpurify (https://www.herbpurify.com/, accessed on 1 April 2024) and Chengdu Must (http://www.cdmust.com/index.aspx, accessed on 1 April 2024) databases.

### 4.2. Cell Cultures

Human keratinocyte cell lines HaCaT and RAW264.3 and Caco-2 cell lines were purchased from the American Type Culture Collection (ATCC, Manassas, VA, USA). During culturing, cells were supplied with DMEM culture medium and supplemented with 10% FBS and 1% penicillin/streptomycin (100 U/mL and 100 μg/mL). The cells were kept in a humidified CO_2_ incubator under a condition of 5% CO_2_ and 37 °C. HaCaT cells were subjected to subculture when cell confluency exceeded 80%.

### 4.3. Cell Viability

Cell viability was assessed with the MTT assay to evaluate the potential toxicity effect of the tested drugs. HaCaT cells were seeded onto 96-well plates for 24 h prior to drug treatments. After incubations for 24 h, an MTT solution in a final concentration of 0.5 mg/mL was added to each well. Following another 3 h of incubation, DMSO solvent was used to dissolve the purple formazan crystals generated in each well. The absorbance of the samples at 570 nm was measured using a microplate reader (Thermo Fisher Scientific). The cell viability was calculated as follows: cytotoxicity (%) = (experimental value − low control)/(high control − low control) × 100%.

### 4.4. Wound Closure Assay

A wound closure assay was performed on cultured HaCaT cells to study cell migration in vitro. Procedures were described previously by Guo et al. [36]. Briefly, HaCaT cells were seeded onto a 12-well plate with a density of 3 × 10^5^ cells/well. Treatments were given when cells grew to full confluency. After serum starvation, sterile 200 μL pipette tips were used to make a vertical and a horizontal scrape on the cell monolayer in a consistent manner. Cells were washed with PBS and treated with drugs at various concentrations. Photos of each cell scrape at 0 (At_0_) and 20 h (At_20_) were captured with a microscope and imaging software (Zen) at a 10× magnification. The software TScratch (CSE lab, Zurich, Switzerland, https://github.com/cselab/TScratch, accessed on 1 September 2024) was used to analyze the open area of each scrape photo at different timepoints. Quantification of the recovery rate of the wound was calculated based on the following equation: wound closure (%) = (At_0_ − At_20_)/At_20_ × 100%.

### 4.5. Computational Docking Study

The docking study was conducted as previously described [37]. Briefly, the chemical structures were generated from Chemdraw (version 20.0, https://revvitysignals.com/products/research/chemdraw accessed on 11 April 2024), and the VEGF protein structure was downloaded from the Protein Data Bank (PDB code: 1FLT, https://www.rcsb.org/, accessed on 11 April 2024). Residues 1-165 of VEGF were previously identified as a key domain responsible for binding to its receptor and were selected as the target domain in the binding stimulation [38]. Virtual screening was performed using SEESAR software (Version 13.0; https://www.biosolveit.de/, accessed on 11 April 2024), and the prediction of ADMET properties, including values of TPSA, Log *p*, HIA and so on, was performed in Optibrium mode.

### 4.6. Stability Test Through HPLC Analysis

The stability test was validated by determining the intra-day and inter-day variability through HPLC analysis, which was performed on an Agilent 1200 Liquid Chromatography (Santa Clara, CA, USA) equipped with an ODS C18 column (4.6 mm × 250 mm). Glycine buffer (pH 8–11), PBS (pH 7–8) and acetate buffer (pH 4–6) were employed to cover main pH ranges. Stock solutions at a concentration of 10 mM of the test compounds were prepared in DMSO and stored at −20 °C, while working concentrations were prepared at 1–5 μM solution in DMSO and buffers. Chromatography was conducted through the ORBAX XDB-C18 column (Agilent, 4.6 × 50 mm, 1.8 μm). The mobile phase was a mixture of water and acetonitrile (95:5, *v*/*v*), and the elution time was 15 min. The mobile phase flow rate and injection volume were 1.0 mL/min and 10 μL, respectively. Analytes were detected at a 210 nm wavelength. The intra-day and inter-day stability was calculated by analyzing six replicates of the standard solution of the two analytes during a single day and six replicates of the samples detected on six consecutive days, respectively. Thereafter, the relative standard deviation (RSD) was determined as a measurement of stability.

### 4.7. Transepithelial Permeability of Chemical Transport

Caco-2 cells were cultured until fully differentiated after being cultured for 21 days. The transepithelial permeability assay was conducted as previously reported [39]. The integrity of the cell monolayer was determined by the transepithelial electrical resistance (TEER) using an EVOM Epithelial Volt/Ohm Mete (WPI, Sarasota, FL, USA) and the permeability of lucifer yellow (a paracellular leakage marker) across the cell monolayer. Prior to drug treatments, the inserts were cleaned twice and equilibrated for 30 min using pre-warmed Hank′s balanced salt solution (HBSS, pH 6.0 at the apical side, pH 7.4 at the basolateral side). The transepithelial permeabilities of the analytes were measured by mass spectrometry. The permeability coefficient (*P*_app_) value was calculated as follows: $P_{app}=\left(\frac{dQ}{dt}\right)\times \frac{1}{C}\times \frac{1}{A}\;$, where (dQ/dt) is the slope of cumulative concentrations of analytes after being received over timepoints and C is the initial concentration of analytes, while A is the surface area of the membrane, which was deemed to be 1.12 cm^2^ here.

### 4.8. Ultrafiltration-Based Affinity Assay

The binding affinity was evaluated through the previous approach [37]. Specifically, the tested ligands at 0.02 µM were mixed with or without 0.02 µM of the VEGF protein (MCE, Monmouth Junction, NJ, USA) and incubated in Milli-Q water (Burlington, MA, USA) at 4 °C for 2 h. The mixture was transferred to a 0.5 mL ultrafiltration tube (2000 MW cutoff; Sartorius Stedim Biotech, Gottingen, Germany). After multiple rounds of centrifugation, the supernatant was further analyzed through HPLC investigation, as described above in Section 4.6.

### 4.9. Luciferase Activity of NF-κB in RAW 264.3

The luciferase of NK-κB activity was performed following a previous report [37]. Briefly, RAW 264.3 cells were transfected with pNF-κB (Thermo Fisher Scientific) using jetPRIME and incubated for 24 h. The refresh medium was replaced every four hours, and drug treatments were added twice after 24 h or 48 h. Upon completion of the reaction, the chemical luminescent intensity was determined by a luminometer (Promega, WI, USA) and normalized by protein concentrations.

## 5. Conclusions

VEGF has been related to various diseases, and anti-VEGF targeted therapy has been identified as an effective strategy in developing treatments against these diseases. Despite the fact that more and more VEGF inhibitors have been discovered over the past few decades, there is still a considerable demand for designing more VEGF-targeted antagonists with higher efficacy and fewer adverse effects. In this study, we conducted intensive drug screening of flavonoids through computational docking analysis and cell models and identified farrerol, ononin and (−)-epicatechin as potential anti-VEGF agents. In future work, comprehensive animal investigations could be employed to further elaborate the pharmacokinetic and pharmacodynamic properties of these phytochemicals. Additionally, structure–activity–relationship studies could be considered a toolbox to modify the chemical scaffold and subsequently improve the corresponding efficacy as well as reduce potential toxicity.

## Figures and Tables

**Table 1:** Chemical stability test of farrerol, ononin and (-)-epicatechin. Analytes were detected at 210 nm wavelength. ^a^ The intra-day precision was determined by analyzing the prepared samples on the same day, *n* = 6; ^b^ the inter-day precision was determined by analyzing the prepared samples on 6 consecutive days, *n* = 6; ^c^ mean peak area is the average peak area of the six chromatograms.

Analytes	Precision			
	Intra-Day (*n* = 6) ^a^		Inter-Day (*n* = 6) ^b^	
	Mean Peak Area ^c^	RSD (%)	Mean Peak Area ^c^	RSD (%)
Farrerol (acidic)	31,803.7	4.09	38,145.2	5.89
Farrerol (neutral)	31,542.0	4.79	36,478.1	4.43
Farrerol (basic)	33,332.4	2.81	35,412.4	6.54
Ononin (acidic)	254,398.2	1.93	275,981.3	4.12
Ononin (neutral)	313,200.4	1.61	265,877.4	3.65
Ononin (basic)	273,422.8	3.12	274,514.7	4.78
Epicatechin (acidic)	2751.5	3.98	2510.4	11.91
Epicatechin (neutral)	2826.6	1.38	2601.7	7.56
Epicatechin (basic)	2726.0	4.85	2519.4	13.58

## Data Availability

The data presented in this study are available upon request from the corresponding author.

## Supplementary Materials

The following supporting information can be downloaded at: https://www.mdpi.com/article/10.3390/molecules30020257/s1, Table S1: Summary of top 20 flavonoid-type phytochemicals from docking analysis; Figure S1: Protein–ligand interactions between VEGF and farrerol (A), (−)-epicatechin (B) and ononin (C). Figures were generated from Discovery Studio 2024 (https://discover.3ds.com/, accessed on 15 October 2024); Figure S2: Cell viability of farrerol, ononin and (−)-epicatechin. HaCaT cells were seeded onto 96-well plates for 24 h prior to drug treatments. Figure S3: Initial screening of other flavonoid-type phytochemicals through wound healing assay in HaCaT cells. All three samples were tested at concentrations of 1, 10, 25, 50 and 100 µM (*n* = 4).

## References

[1] Mandal K., Kent S.B.H. (2011). Total chemical synthesis of biologically active vascular endothelial growth factor. Angew. Chem. Int. Ed..

[2] Shaw P., Dwivedi S.K.D., Bhattacharya R., Mukherjee P., Rao G. (2024). VEGF signaling: Role in angiogenesis and beyond. Biochim. Biophys. Acta Rev. Cancer.

[3] Apte R.S., Chen D.S., Ferrara N.F. (2019). VEGF in signaling and disease: Beyond discovery and development. Cell.

[4] Park S.A., Jeong M.S., Ha K.-T., Jang S.B. (2018). Structure and function of vascular endothelial growth factor and its receptor system. BMB Rep..

[5] Kanellies J., Fraser S., Katerelos M., Power D.A. (2000). Vascular endothelial growth factor is a survival factor for renal tubular epithelial cells. Am. J. Physiol. Renal. Physiol..

[6] Noel A., Jost M., Lambert V., Lecomte J., Rakic J.-M. (2007). Anti-angiogenic therapy of exudative age-related macular degeneration: Current progress and emerging concepts. TRENDS Mol. Med..

[7] Panche A.N., Diwan A.D., Chandra S.R. (2016). Flavonoids: An overview. J. Nutr. Sci..

[8] Chen L., Yang B., Tang B., Gong G., Kam H., Gao C., Chen Y., Wang R., Lee S.M.-Y. (2018). Differential angiogenic activities of naringin and naringenin in zebrafish in vivo and human umbilical vein endothelial cells in vitro. J. Funct. Foods.

[9] Zhou Z., Mao W., Li Y., Qi C., He Y. (2019). Myricetin inhibits breast tumor growth and angiogenesis by regulating VEGF/VEGFR2 and p38MAPK signaling pathways. Anatonical Rec..

[10] Lin X., Li X., Lin X. (2020). A review on applications of computational methods in drug screening and design. Molecules.

[11] Kelly R.J., Rixe O. (2009). Axitinib—A selective inhibitor of the vascular endothelial growth factor (VEGF) receptor. Targ. Oncol..

[12] Beneta L.Z., Hoseya C.M., Ursub O., Opreaba T.I. (2016). BDDCS, the rule of 5 and drugability. Adv. Drug Deliv. Rev..

[13] Ali J., Camilleri P., Brown M.B., Hutt A.J., Kirton S.B. (2012). Revisiting the general solubility equation: In silico prediction of aqueous solubility incorporating the effect of topographical polar surface area. J. Chem. Inf. Model..

[14] Hou T. (2007). ADME evaluation in drug discovery. 8. The prediction of human intestinal absorption by a support vector machine. J. Chem. Inf. Model..

[15] Wu D., Chen Q., Chen X., Han F., Chen Z., Wang Y. (2023). The blood–brain barrier: Structure, regulation, and drug delivery. Nature.

[16] Garrido A., Lepailleur S., Mignani S.M., Dallemagne P., Rochai C. (2020). hERG toxicity assessment: Useful guidelines for drug design. Eur. J. Med. Chem..

[17] Juvale I.I.A., Hamid A.A.A., Halim K.B.A., Has A.T.C. (2022). P-glycoprotein: New insights into structure, physiological function, regulation and alterations in disease. Heliyon.

[18] Blessy M., Ruchi D.P., Prajesh N.P., Agrawal Y.K. (2014). Development of forced degradation and stability indicating studies of drugs—A review. J. Pharm. Anal..

[19] Ahmed I., Leach D.N., Wohlmuth H., De Voss J.J., Blanchfield J.T. (2020). Caco-2 cell permeability of flavonoids and saponins from Gynostemma pentaphyllum: The immortal herb. ACS Omega.

[20] Dabravolski S.A., Khotina V.A., Omelchenko A.V., Kalmykov V.A., Orekhov A.N. (2022). The role of the VEGF family in atherosclerosis development and its potential as treatment targets. Int. J. Mol. Sci..

[21] Tu J., Fang Y., Han D., Tan X., Jiang H., Gong X., Wang X., Hong W., Wei W. (2021). Activation of nuclear factor-κB in the angiogenesis of glioma: Insights into the associated molecular mechanisms and targeted therapies. Cell Prolif..

[22] Melincovici C.S., Boşca A.B., Şuşman S., Mărginean M., Mihu C., Istrate M., Moldovan I.M., Roman A.L., Mihu C.M. (2018). Vascular endothelial growth factor (VEGF)—Key factor in normal and pathological angiogenesis. Rom. J. Morphol. Embryol..

[23] Fong G.H., Rossant J., Gertsentein M., Breitman M.L. (1995). Role of the Flt-1 receptor tyrosine kinase in regulating the assembly of vascular endothelium. Nature.

[24] Liu Y., Li Y., Wang Y., Lin C., Zhang D., Chen J., Ouyang L., Wu F., Zhang J., Chen L. (2022). Recent progress on vascular endothelial growth factor receptor inhibitors with dual targeting capabilities for tumor therapy. J. Hematol. Oncol..

[25] Ghalehbandi S., Yuzugulen J., Pranjol Z.I., Pourgholami M.H. (2023). The role of VEGF in cancer-induced angiogenesis and research progress of drugs targeting. Eur. J. Pharmacol..

[26] U.S. Food and Drug Administration. https://www.fda.gov/.

[27] Ferrara N., Hillan K.J., Gerber H.-P., Novotny W. (2014). Discovery and development of bevacizumab, an anti-VEGF antibody for treating cancer. Nat. Rev. Drug. Discov..

[28] Escalante C.P., Zalpour A. (2011). Vascular endothelial growth factor inhibitor-induced hypertension: Basics for primary care providers. Cardiol. Res. Pract..

[29] Hu W.-H., Duan R., Xia Y.-T., Xiong Q.-P., Wang H.-Y., Chan G.K.-L., Liu S.-Y., Dong T.T.-X., Qin Q.-W., Tsim K.W.-K. (2019). Binding of resveratrol to vascular endothelial growth factor suppresses angiogenesis by inhibiting the receptor signaling. J. Agric. Food Chem..

[30] Hu W.-H., Chan G.K.-L., Wang H.-Y., Kong X.-P., Dong T.T.-X., Tsim K.W.-K. (2019). Synergy of ginkgetin and resveratrol in suppressing VEGF-induced angiogenesis: A therapy in treating colorectal cancer. Cancers.

[31] Khater M., Greco F., Osborn H.M.I. (2020). Antiangiogenic activity of flavonoids: A systematic review and meta-analysis. Molecules.

[32] Subbaraj G.K., Kumar Y.S., Kulanthaivel L. (2021). Antiangiogenic role of natural flavonoids and their molecular mechanism: An update. Egypt. J. Intern. Med..

[33] Wei Q., Zhang Y.H. (2024). Flavonoids with anti-angiogenesis function in cancer. Molecules.

[34] Ramanan M., Sinha S., Sudarshan K., Aidhen I.S., Doble M. (2016). Inhibition of the enzymes in the leukotriene and prostaglandin pathways in inflammation by 3-aryl isocoumarins. Eur. J. Med. Chem..

[35] Sudarshan K., Aidhen I.S. (2017). Convenient Synthesis of 3-Glycosylated Isocoumarins. Eur. J. Org. Chem..

[36] Guo M.S., Gao X., Hu W., Wang X., Dong T.T.-X., Tsim K.W.-K. (2022). Scutellarin potentiates the skin regenerative function of self-growth colony, an optimized platelet-rich plasma extract, in cultured keratinocytes through VEGF receptor and MAPK signaling. J. Cosmet. Dermatol..

[37] Gao A.X., Xia T.C.-X., Mak M.S.-H., Kwan K.K.-L., Zheng B.Z.-Y., Xiao J., Dong T.T.-X., Tsim K.W.-K. (2021). Luteolin stimulates the NGF-induced neurite outgrowth in cultured PC12 cells through binding with NGF and potentiating its receptor signaling. Food Funct..

[38] Zhao W., McCallum S.A., Xiao Z., Zhang F., Linhardt R.J. (2012). Binding affinities of vascular endothelial growth factor (VEGF) for heparin-derived oligosaccharides. Biosci. Rep..

[39] Zheng K.Y.-Z., Choi R.C.-Y., Guo A.J.-Y., Bi C.W.-C., Zhu K.Y., Du C.Y.-Q., Zhang Z.-X., Lau D.T.-W., Dong T.T.-X., Tsim K.W.-K. (2012). The membrane permeability of astragali radix-derived formononetin and calycosin is increased by angelicae sinensis radix in caco-2 cells: A synergistic action of an ancient herbal decoction Danggui Buxue Tang. J. Pharm. Biomed. Anal..

