# Supplementary material for: Drug Screening of Flavonoids as Potential VEGF Inhibitors Through Computational Docking and Cell Models
Source: Molecules. 2025 Jan 10;30(2):257. doi: 10.3390/molecules30020257 (PMC11767819; doi:10.3390/molecules30020257)
Supplement: Supplementary file 1 [file molecules-30-00257-s001.zip › molecules-3391093-supplementary/molecules-3391093-supplementary.pdf]

Supplementary Materials

Drug Screening of Flavonoids as Potential VEGF Inhibitors Through Computational Docking and Cell Models

Shengying Lin [1,2], Roy Wai-Lun Tang [1,2], Yutong Ye [1,2], Chenxi Xia [1,2], Jiahui Wu [1,2], Ran Duan [1,2], Ka-Wing Leung [1,2], Tina Ting-Xia Dong [1,2] and Karl Wah-Keung Tsim [1,2,*]

[1] Center for Chinese Medicine, Division of Life Science, The Hong Kong University of Science and Technology, Clear Water Bay, Kowloon, Hong Kong, China; lishlin@ust.hk (S.L.); roytwl@ust.hk (R.W.-L.T.); yyebl@connect.ust.hk (Y.Y.); chenxi.xia@connect.ust.hk (C.X.); jwuct@connect.ust.hk (J.W.); duanran@ust.hk (R.D.); lkwing@ust.hk (K.-W.L.); botina@ust.hk (T.T.-X.D.)
[2] State Key Laboratory of Molecular Neuroscience, Division of Life Science, The Hong Kong University of Science and Technology, Clear Water Bay, Kowloon, Hong Kong, China
* Correspondence: botsim@ust.hk; Tel.: +852-2358-7332

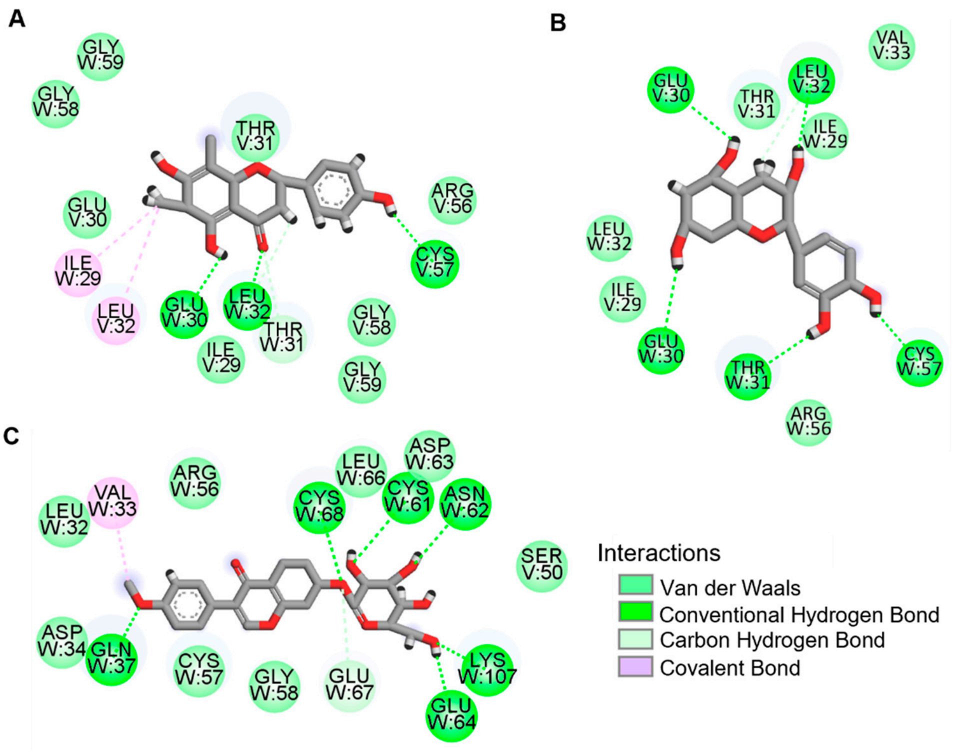

Figure S1. Protein-ligand interactions between VEGF and farrerol (**A**), (−)-epicatechin (**B**) and ononin (**C**). Figures were generated from Discovery Studio 2024 (https://discover.3ds.com/, accessed on 15 October 2024).

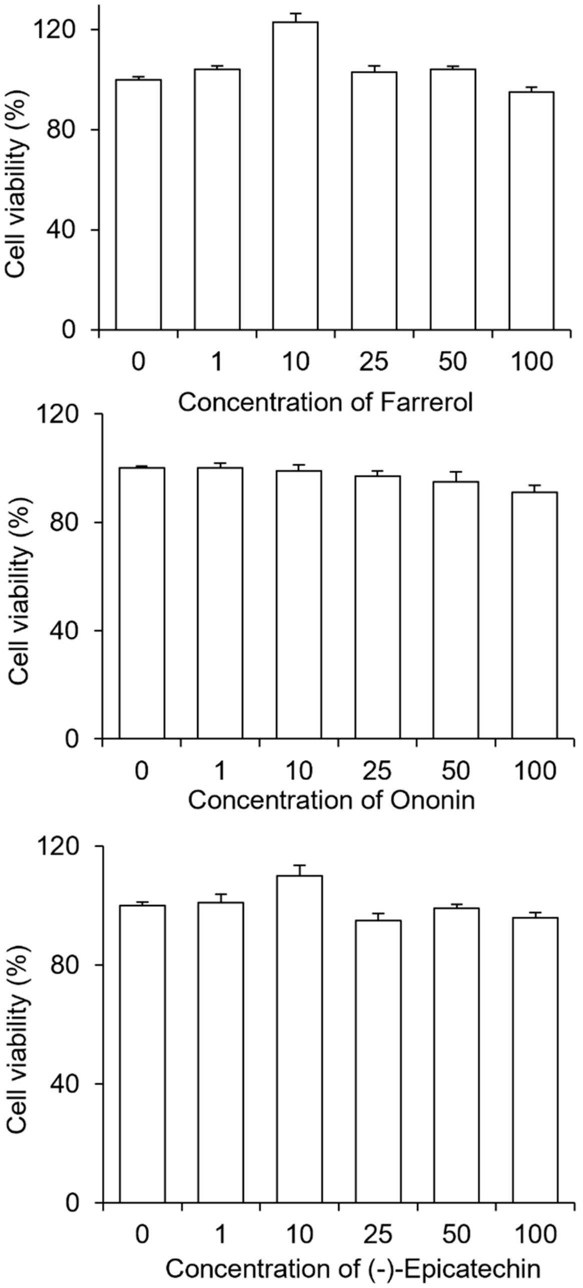

Figure S2. Cell viability of farrerol, ononin and (−)-epicatechin. HaCaT cells were seeded onto 96-well plates for 24 h, prior to drug treatments. All three samples were tested at concentrations of 1, 10, 25, 50 and 100 μM (*n* = 4).

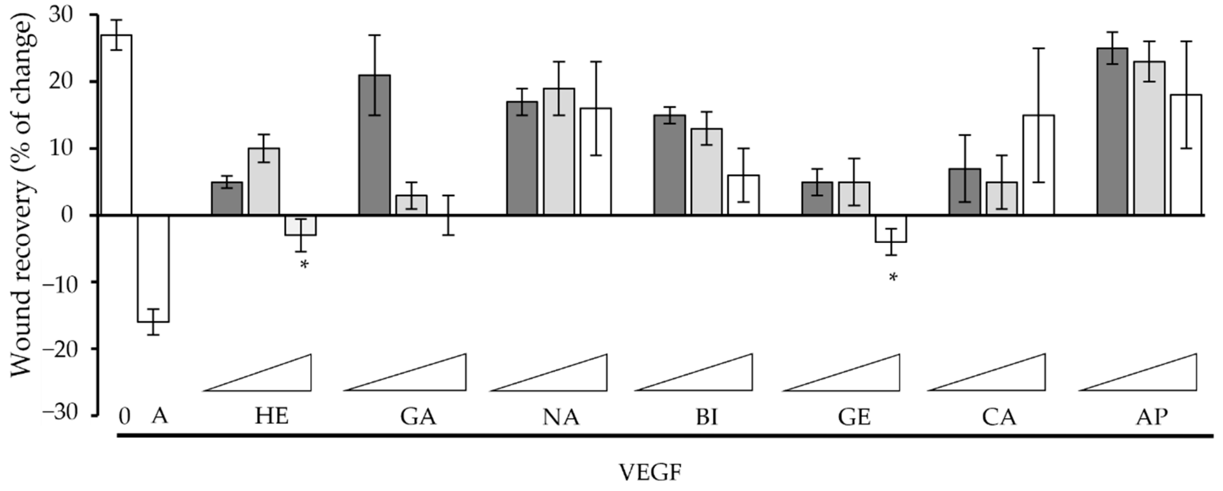

Figure S3. Initial screening of other flavonoid-type phytochemicals through wound healing assay in HaCaT cells. All three samples were tested at concentrations of 1, 10, 25 μM. The data indicated the mean ± SD (n = 4), as percentage of change compared with the blank group, and the asterisks represented the statistically significant differences such that * p < 0.05, compared with the blank group. A: avastin; HE: hesperetin; GA: galangin; NA: naringenin; BI: biochanin A; GE: genistein; CA: calycosin; AP: apigenin.
